# Syndromic Disorders with Short Stature

**DOI:** 10.4274/Jcrpe.1149

**Published:** 2014-03-05

**Authors:** Zeynep Şıklar, Merih Berberoğlu

**Affiliations:** 1 Ankara University School of Medicine, Department of Pediatric Endocrinology, Ankara, Turkey

**Keywords:** short stature, Noonan syndrome, Prader-Willi syndrome, Aarskog syndrome, Silver-Russell syndrome

## Abstract

Short stature is one of the major components of many dysmorphic syndromes. Growth failure may be due to a wide variety of mechanisms, either related to the growth hormone (GH)/insulin-like growth factor axis or to underlying unknown pathologies. In this review, the relatively more frequently seen syndromes with short stature (Noonan syndrome, Prader-Willi syndrome, Silver-Russell syndrome and Aarskog-Scott syndrome) were discussed. These disorders are associated with a number of endocrinopathies, as well as with developmental, systemic and behavioral issues. At present, GH therapy is used in most syndromic disorders, although long-term studies evaluating this treatment are insufficient and some controversies exist with regard to GH dose, optimal age to begin therapy and adverse effects. Before starting GH treatment, patients with syndromic disorders should be evaluated extensively.

## INTRODUCTION

The term “syndrome” refers to a group of specific features which appear to be unrelated, but which define a number of disorders when they develop together. Children with dysmorphic features may be patients with a syndrome associated with a chromosal abnormality, low birth, mental retardation and short stature (1). There are very many dysmorphic syndromes with short stature as a component. Short stature may be either proportionate or disproportionate. In this review, we discuss the relatively more frequently seen syndromes, i.e. Noonan syndrome (NS), Prader-Willi syndrome (PWS), Silver-Russell syndrome (SRS) and Aarskog-Scott syndrome (ASS), which all have severe or moderate short stature as a common feature. Turner syndrome (TS) will not be taken up in this paper, because it has been extensively discussed in previous publications. There are more than 200 different skeletal dysplasia which can be suspected in patients with disproportionate short stature, but these also have not been included in this review.

In most of syndromic disorders, the cause of short stature is based at the cellular level. Growth failure as part of many syndromes may be due to a wide variety of mechanisms and in many of these syndromes, the underlying mechanisms are still unknown. Usually, there is no growth hormone (GH) GH deficiency (GHD), but in some patients, a pathology in the GH/insulin-like growth factor-1 (GH/IGF-1) axis can be detected (1). In recent years, recombinant human GH (rhGH) treatment has been introduced in several syndromic disorders with short stature, regardless of their GH status, while in some syndromes, such as Bloom syndrome which has high rate of chromosomal breakage and a high risk of malignancy, rhGH has not been advocated (1). The syndromic disorders with severe and moderate short stature are listed in Table 1 (2).

## NOONAN SYNDROME

NS (OMIM163950) is one of the most commonly encountered syndromes with Mendelian inheritance. It has an estimated incidence of 1/1000 to 1/2500 live births (3). The specific facial features (hypertelorism, ptosis, down-slanting palpebral fissures, low-set posteriorly rotated ears), short stature, congenital heart defects (pulmonary valve stenosis, hypertrophic cardiomyopathy, atrial septal defect), as well as chest and spinal deformities are the typical signs of NS. Mild mental retardation, learning disabilities, feeding difficulties in infancy, cerebrovascular abnormalities, abnormal pigmentation, cryptorchidism, lymphedema, coagulation defects and hearing defects are frequently seen in patients with NS (3,4,5).

There is no gender difference in frequency. Nearly 20% of the cases are familial, most showing an autosomal dominant inheritance (6).

The majority of boys diagnosed with NS have cryptorchidism either unilaterally or bilaterally. Primary Sertoli cell dysfunction is also seen in male NS patients, which is the cause of testicular dysfunction and low fertility. In female NS patients, fertility is not impaired (5).

**Figure 1:**
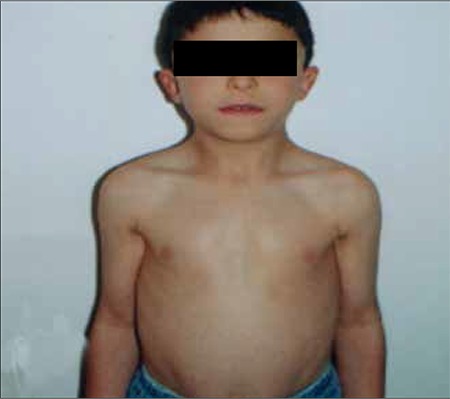
Phenotype of a patient with Noonan syndrome

The diagnosis of NS is mainly based on clinical evaluation. The typical facial features and cardiac pathology are usually the signs leading to consideration of a diagnosis of NS (Figure 1). The facial features may not be distinctive in the neonatal period. However, the diagnosis of NS is suspected in newborn infants with generalized edema, webbed neck and congenital heart disease. The facial features can vary within the same family. With increasing age, clinical features become most discrete and are more subtle in adulthood (3,4,5).

The diagnostic criteria for NS were proposed by Van der Burght in 1994 and have been used extensively since (Table 2). According to these criteria, definitive diagnosis of NS is based on presence of: [1] “typical face dysmorphology + one major sign or two minor signs”, or [2] “suggestive face dysmorphology + two major or three minor signs” (7).

**Genetics**

NS is related to changes in the RAS/RAF- mitogen activated protein-kinase (MAPK) signaling pathway, which is implicated in growth factor-mediated cell proliferation, differentiation and apoptosis (3,8).

There are several syndromes whose phenotypes significantly overlap with NS. These syndromes are also related to the RAS/RAF-MAPK pathway and include Cardio-facio-cutaneous (CFC) syndrome (OMIM115150), Costello syndrome (OMIM218040), neurofibromatosis type 1 (OMIM162200) and Leopard syndrome (OMIM151100).

NS girls can be misdiagnosed as TS because these two syndromes have clinically similar features (6). A karyotype analysis is needed to make the differential diagnosis.

The Leopard syndrome is inherited in an autosomal dominant pattern and the term is acronym for multiple Lentigenes, Electrocardiographic conduction abnormalities, Ocular hypertelorism, Pulmonary stenosis, Abnormal genitalia, Retardation of growth and sensorineural Deafness (9). In young children, the clinical features of the Leopard syndrome are very similar to NS. Café-au-lait spots which appear in early infancy and generalized multiple lentigines after 5-6 years of age are the main distinguishing characteristics of this syndrome.

The Costello syndrome and CFC syndrome have similarities with NS, although in comparison with NS, the growth retardation is more severe and the patients have coarser facial features in these syndromes (6). Patients with Costello syndrome generally have macrocephaly, cutis laxa, nasal and perioral papillomata, deep palmar and plantar creases, diffuse skin hyperpigmentation, nail dysmorphology and increased risk of rhabdomyosarcoma. CFC syndrome patients usually have ectodermal abnormalities such as sparse hair and eyebrows, follicular hyperkeratosis, palmoplantar hyperkeratosis and an ichthyosis-like condition (6).

PTPN11, SOS1, KRAS, NRAS, BRAF1, SHOC2 and CBL are the genes in the RAS-MAPK signaling pathway which cause NS or related conditions (5). In 2001, Tartaglia et al (10), for the first time, identified a heterozygous missense mutation in the PTPN11 gene, a gene mapped to chromosome 12q24.1, in a patient with NS. Following this report, further studies confirmed PTPN11 as the most affected gene in NS patients. It was demonstrated that 29-60% of NS cases are caused by mutations in PTPN11 gene (5,11). PTPN11 gene-encoded SHP2, as a non-receptor protein-tyrosine phosphatase, is involved in the regulation of the phosphotyrosine content of specific intracellular proteins (9).

Specific somatic PTPN11 mutations were also found in juvenile myelomonocytic leukemia and in several human cancer patients. This observation raises the speculation that somatic mutations in components of this pathway might play a role in the pathogenesis of cancer. However, some aminoacid substitutions are preferentially associated with NS or cancer (5). It was reported that germline mutations that cause NS are less able to cause SHP2 gain of function than do the somatic mutations associated with leukemia (12). In addition, the incidence of cancer in patients with NS has not been shown to be increased over that of the general population (4).

**Growth**

Growth retardation is a major criterion for the clinical diagnosis of NS, adult height being about 152 cm for females and 162 cm for males (13). Growth pattern may also carry distinctive characteristics. At birth, weight and height are within normal limits. In the first year of life, there is a rapid decline in height standard deviation score (SDS). After 2-4 years of age, mean height follows the 3rd percentile until about 12 years in males and 10 years in females. Puberty is delayed by about two years with a low peak height velocity (14).

Normal responses are usually obtained in GH stimulation tests, but IGF-1 levels are found to be low and spontaneous GH secretion is impaired (13). It has been suggested that defective signaling of RAS-MAPK pathway is the cause of the growth disturbances seen in NS. When short-term studies showed that rhGH therapy improved growth velocity in NS, long-term studies were undertaken. Final height in patients with NS treated with GH showed a height gain of 0.6 to 2.0 SDS over the controls. The benefit of GH treatment seems to be less marked in patients with PTPN11 mutation, suggesting a mode of GH insensitivity (11,15). There is a need for larger prospective studies to conclude whether the PTPN11 gene mutation or other mutations correlate with the growth response to GH treatment and also to assess the magnitude of height gain.

## PRADER-WILLI SYNDROME

**Figure 2:**
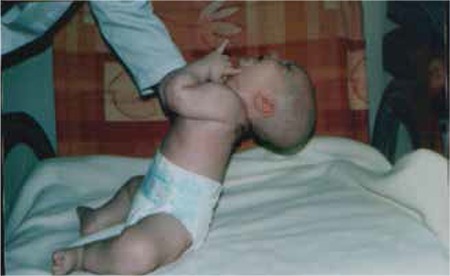
Hypotonia in an infant with Prader-Willi syndrome

WS is a rare neurogenetic disorder characterized by short stature, muscular hypotonia, abnormal body composition, progressive obesity, hypogonadism, mental retardation, behavioral abnormalities, respiratory and sleep disturbances and dysmorphic features (16). Its prevalence was reported as 1/10 000 to 1/30 000 live births (17). The birth weight and length of infants with PWS is 15-20% smaller than their unaffected siblings. Hypotonia is one of the main characteristics and its first clues are decreased fetal movement or delivery difficulties. In the infancy period, hypotonia is a universal finding and improves over time (Figure 2). Motor development and language milestones are delayed in the majority of children with PWS. Intellectual and/or learning disabilities become evident as the child grows older. Obesity is a major problem in these children. Excessive weight gain, hyperphagia and decreasing of satiety begin in early childhood. Characteristic facial features, strabismus, small hands and feet, as well as scoliosis are often present (17). The consensus clinical diagnostic criteria of PWS were released in 1993 and then revised (Table 3) (18).

**Genetics**

The mainstay of diagnosis is DNA testing. Absence of expression of one of the paternally inherited genes localized in chromosome 15q11-q13 region leads to PWS. This disorder is an imprinted condition and DNA-based methylation testing will detect abnormal imprinting in 99% of affected individuals. Fluorescence in situ hybridization or chromosomal microarray is also available techniques to diagnose patients who have deletion of chromosome 15q11.2-q13. Approximately 70% of the cases have a de novo deletion in the paternally inherited chromosome 15q11-q13 region. The remaining cases occur as a result of a maternal uniparental disomy (UPD) of chromosome 15 (20-25%) or as a result of either microdeletions or epimutations in the 15q11-q13 region (2-5%) (17,19). No single gene mutation has been found that explains all the features of PWS. Although the exact function of each of the genes responsible for the PWS phenotype remains to be elucidated, Necdin is one of the genes inactivated in children with PWS who are predisposed to increased adiposity at the expense of lean mass (20,21).

**Growth**

Short stature is usually seen in PWS patients and specific growth charts have been developed. Most patients have hypogonadism and GHD, leading to short stature, probably caused by hypothalamic dysfunction (22,23). Within the first two years of life, the height of these patients was shown to decrease to below the 3rd centile. Subsequently, growth rate shows a mild improvement and height measurements persist in the 10th centile zone until age 10 to 12 years. After 12-14 years of age, the percentile values again decrease below the 5th centile. In untreated individuals, mean final height was reported as 155 cm for males and 148 cm for females (17,18,22). Short stature is caused by GHD and by absence of a pubertal growth spurt. Eighty percent of children with PWS have GHD, which is also seen in 50% or more of adult patients. Data from several studies document reduced GH response to GH stimulation tests, low serum IGF-1 and IGF binding protein-3 levels and decreased 24-hour spontaneous GH release in PWS patients (17,18).

Features such as reduced muscle strength, altered body composition, obesity, low energy expenditure, short stature, abdominal obesity, delayed bone age seen in PWS resemble the features of GHD (24).

GH therapy is an approved treatment option for children with PWS and the beneficial effects of GH treatment on growth and body composition have been clearly demonstrated. GH treatment has been shown to increase growth rate and muscle mass and to decrease body fat mass (18,25,26).

In infants with PWS, several beneficial effects of GH therapy, such as increase in head circumference as well as improvement in gross motor skills, in behavior indices, in cognitive function and in language acquisition, have also been reported (18,25,26). Respiratory metabolic rate has been shown to be higher in treated children than in non-treated ones (23).

There are recent studies which show the long-term effect of GH therapy in PWS. Children with PWS treated with GH through childhood are able to achieve their expected midparental height (27). In one study, the effects of GH treatment in 22 children with genetically verified PWS were followed from the start of treatment to near-adult height. These patients reached a near-adult height within the midparental height median of -0.5 SDS and of +0.9 SDS for boys and girls, respectively and it was concluded that GH treatment in children with PWS normalizes adult height (16,28).

Furthermore, cognitive impairment is not accepted as a barrier to GH treatment (24). GH treatment can influence the respiratory functions. Lindgren et al (26) reported that GH treatment of children with PWS has marked stimulatory effects on ventilation and normalizes in part the abnormal CO2. On the other hand, the significance of respiratory disorders has been highlighted in children with PWS during initiation of rhGH (24,28,29).

There are concerns about an increased risk of death in children with PWS receiving GH therapy (30,31). The relationship of GH administration to unexpected death remains unclear. However, there are also several studies which report that the rate of death in patients with PWS on and off GH did not differ (32,33). Actually, children with PWS have a high incidence of both central apnea and obstructive apnea. Severe obesity or intercurrent asymptomatic respiratory tract infection can exacerbate obstructive apnea and may even lead to sudden death. It has been recommended that GH therapy should not be initiated in children with breathing difficulties or during an acute respiratory infection (24).

Scoliosis is another problem frequently seen in children with PWS (40-80%). There is a concern about whether GH treatment would increase the frequency or severity of this finding. Until now, no relationship was found between GH therapy and the age of onset or severity of scoliosis in children with PWS (34). No side effects have been reported regarding the metabolic status of children with PWS given GH therapy.

**Other Specific Problems**

In patients with PWS, there is an altered body composition with increased body fat mass and decreased lean mass. Energy expenditure and resting metabolic rate are also decreased (23).

The increase in body fat mass is extreme and the percentage of body fat in PWS patients was reported to be greater than that in obese individuals (25,35).

Obesity is a major problem and frequently leads to development of type 2 diabetes, hypertension, dyslipidemia, cardiopulmonary failure, sleeping disturbances and respiratory problems. Hypotonia may lead to scoliosis. Obstructive sleep apnea is also a frequent finding (24,25,29).

In PWS patients, glucose metabolism is often normal in childhood. Diabetes mellitus starts at a mean age of 20 years. Insulin sensitivity and risk of metabolic syndrome may vary depending upon degree of obesity, body fat distribution, genetic background and medication (17). To monitor for potential side effects of GH treatment on glucose metabolism, HbA1c, fasting glucose and serum insulin levels need to be evaluated at three-month intervals in all patients with PWS who are older than twelve years and at an earlier age if they have a family history of diabetes (24).

Hypogonadism occurs in both female and male PWS patients. Genital hypoplasia, pubertal development disorders and infertility are encountered in these patients. Although the genital hypoplasia is often overlooked in females, micropenis, hypoplastic scrotum, or cryptorchidism are detected frequently in males. The hypogonadism in PWS is due to a combination of hypothalamic and primary gonadal deficiencies. Central hypothyroidism has been detected in up to 25% of patients with PWS. The mean age of diagnosis of central hypothyroidism is reported as age 2 years (17).

## SILVER-RUSSELL SYNDROME

SRS (OMIM180860) is characterized by severe intrauterine growth retardation, a reduced postnatal growth rate and clinical features such as a high forehead, preserved head circumference, small jaw, triangular face, clinodactyly, camptodactyly, short stature, hypospadias, skeletal asymmetry, lean body habitus and developmental delay. Price et al (36) described the major features when considering a diagnosis of SRS: 1) birth weight below or equal to -2 SD; 2) poor postnatal growth, below or equal to -2 SD; 3) preservation of occipitofrontal circumference, 4) characteristic facial phenotype; and 5) asymmetry. The incidence has been estimated as from 1 in 3000 to 1 in 100 000 live births (37).

Because the facial features of SRS tend to become less obvious with age, making the clinical diagnosis in adults can be difficult. Asymmetry can be seen in the trunk, face, or limbs. Other frequent clinical features include feeding difficulties in early childhood and excessive sweating in infancy. Congenital anomalies (cleft palate, congenital heart disease, genital anomalies, limb defects), or increased risk of developing myoclonus-dystonia have been reported in a few patients (38).

There is no pathognomonic radiological feature for SRS. However, delayed bone age, clinodactyly, 5th middle or distal phalangeal hypolasia, ivory epiphyses and second metacarpal pseudoepiphysis have been reported as suggestive features (39).

**Genetics**

SRS is a genetically heterogeneous disorder. Autosomal dominant, autosomal recessive and X-linked inheritance models have been reported. Recent findings have shown that loci on chromosome 11 have a major role in this disorder. Up to 50% of SRS patients have methylation defects in the imprinted domain (hypomethylation of the IGF-2/H19 imprinted region) on chromosome 11p15. Ten per cent of patients have maternal UPD for chromosome 7. There are still at least 40% of SRS patients with an unknown genetic etiology (37,40). SRS is the first human disorder associated with epigenetic mutations affecting two different chromosomes (40).

Chromosome 11p15 contains a cluster of imprinted genes, which are important to the control of fetal growth. The region consists of two imprinted control regions (ICR). Interestingly, chromosome 11p15 region is also involved in the aetiology of Beckwith-Wiedemann syndrome (BWS), which has quite different features from SRS. Disturbances of the telomeric ICR1 region and the more centromeric ICR2 region were reported to result in this overgrowth syndrome (38,40).

**Growth**

Children with SRS constitute a very small subgroup of small for gestational age (SGA). Thus, less is known about GH/IGF-1 axis in SRS children than in SGA children without SRS. The majority of patients with SRS have low birth weight (below -2 SDS) and decreased postnatal height. Birth weight is typically 1900-2000 g in term babies. Growth performance is poor in infancy and early childhood. Height SDS is reported as -3.5 and -4 SD by the age of 4 years. Children with SRS show no postnatal catch-up growth. During childhood, height usually remains below the 3rd centile. Bone age is usually delayed. Mean adult height in males was reported as 151.2 cm and in females 139.9 cm (38,39).

Spontaneous GH secretion appear to be impaired. However, the exact contribution of altered growth parameters to severity of short stature has not been demonstrated (39). Children with maternal UPD have a higher birth length but lower postnatal height increment when compared to patients with ICR1 hypomethylation (38). Chromosome 11p15 mutations deregulate IGF2 and CDKN1C genes, which affect normal growth; thus, restrict fetal and early childhood growth (39).

In children with SRS, data on diminished pulse and frequency of GH secretion overnight is limited. IGF1 receptor gene mutations were also not found in a sample of SRS patients (41). RhGH is widely accepted as treatment for SGA, including children with SRS. Although GH treatment is often considered for a child with SRS who has not acquired adequate catch-up growth at the age of 2 years, there are no randomized controlled studies on the effectiveness of GH therapy in SRS (40,42). Data related to long-term GH therapy in patients with SRS are insufficient (39). In a recent study on a group of 26 children with SRS, long-term GH therapy led to a significant improvement in growth with a final height of -1.3 SDS. A greater increment in final height was observed in patients with lower heights at the start of treatment (43). Information about the possible adverse events during GH treatment in children with SRS is also limited. In a study (44), it was shown that GH treatment does not increase skeletal asymmetry. On the other hand, there is concern about development of insulin resistance in children with SRS treated with GH (38).

## AARSKOG-SCOTT SYNDROME (FACIOGENITAL SYNDROME)

ASS (OMIM305400) is a genetically heterogeneous disorder, associated with X-linked, autosomal dominant, or autosomal recessive inheritance (45). Before the first description was made by Aarskog and Scott in 1970, these patients were possibly diagnosed as NS (46,47). This syndrome is also named “faciogenital dysplasia” and is characterized by short stature and facial, limb and genital anomalies. Patients with ASS show considerable phenotypic heterogeneity and symptoms may range from mild to severe. Minor features usually affect the midline and the urogenital system (hypertelorism, umbilical hernia, shawl scrotum, hypospadias, undescended testes), while dysplastic changes involve the skeleton (48,49). In addition to the typical dysmorphology, short stature and mental retardation are also usually seen in ASS. The severity of growth retardation is mild to moderate and disproportionate with acromelia. Mild or moderate mental retardation was reported to occur in frequencies as high as 30% of the patients. However, hyperactivity and attention deficit disorders are even more frequent. The diagnosis of AAS is primarily made on the basis of clinical criteria such as genital anomalies (shawl scrotum, cryptorchidism), brachydactyly and hypertelorism. Clinical findings are summarized in Table 4 (2). Typical facial features include a round face, facial edema in children younger than 4 years, downward slanting palpebral fissures, a short nose with anteverted nares, long filtrum, ocular hypertelorism with ptosis of different degrees, maxillary hypoplasia, a broad upper lip, widow’s peak, a crease below the lower lip, orthodontic problems and abnormal auricules (2,50,51,52). Brachydactyly, clinodactily of the fifth finger, joint laxity, mild interdigital webbing, short broad hands and feet, simian line, bulbous toes are the main skeletal features of ASS. Mild pectus excavatum can also occur (2,51). The facial features in ASS change with age, with hypertelorism becoming less obvious, the forehead becoming less prominent and the face becoming elongated. The typical morphology of the hands (brachydactyly with interdigital webbing and joint laxity) also becomes less evident with age. Cardiac defects, especially pulmonic stenosis or ventricular septal defect have also been described in ASS (50). In a case report, it was suggested that the myopathy also may be part of the clinical stigmata of ASS and that ptosis may be a manifestation of myopathy (49).

Most male patients have shawl scrotum. Cryptorchidism and inguinal hernia may be accompanying features (50).

Failure to thrive with feeding difficulties can occur in the first year of life. Recurrent respiratory infections have been reported in 35% of patients (2).

**Genetics**

As described previously, ASS is a genetically heterogeneous syndrome and a part of X-linked entities caused by mutation of the FGD1 gene mapped to the Xp11.21 region. The FGD1 gene encodes a guanine nucleotide exchange factor (GEF) which is important for signaling pathways involved in cytoskeletal organization, skeletal formation and morphogenesis. Almost 20% of ASS patients have FGD1 gene mutation (53). Despite the feasibility of molecular diagnosis, to date only a limited number of mutations have been reported in ASS patients. This situation either can be related to genetic heterogeneity of the disorder or the overlapping clinical features of ASS with other unrelated conditions, such as NS, pseudohypoparathyroidism and Robinow’s syndrome (53).

**Growth**

Growth failure is one of the main features of ASS and may start prenatally. Growth rate is low during the first year of life and becomes distinct between 1 and 3 years of age. During childhood, growth rate is slow and height usually remains below the 3rd centile. Patients have often delayed puberty but normal fertility. Adult height was reported as -2 and -3 SD (51).

Response to GH stimulation test is usually normal in children with ASS. GH treatment has positive effect on growth and final height. Response to GH treatment was evaluated in 21 patients with ASS enrolled in the KIGS database. After 3 years of GH treatment with a standard dose (0.22 mg/kg/week), a beneficial effect on growth increment was shown (51). There is no randomized controlled studies in this rare syndrome.

In conclusion, syndromic disorders with short stature are associated with a number of endocrinopathies as well as with developmental, systemic and behavioral problems. Growth failure may be associated with aberrations in the GH/IGF-1 axis or may be related to other specific problems. A multidisciplinary team approach is required for evaluation and treatment of these patients. In most of syndromic disorders, GH therapy is widely accepted by clinicians, but some controversies exist with regard to GH dose, optimal age to begin GH therapy and possible adverse effects. Before starting GH treatment, patients should be evaluated extensively with regard to respiratory disturbances, glucose metabolism, malignancy risk and other undesirable effects of this treatment.

## Figures and Tables

**Table 1:**
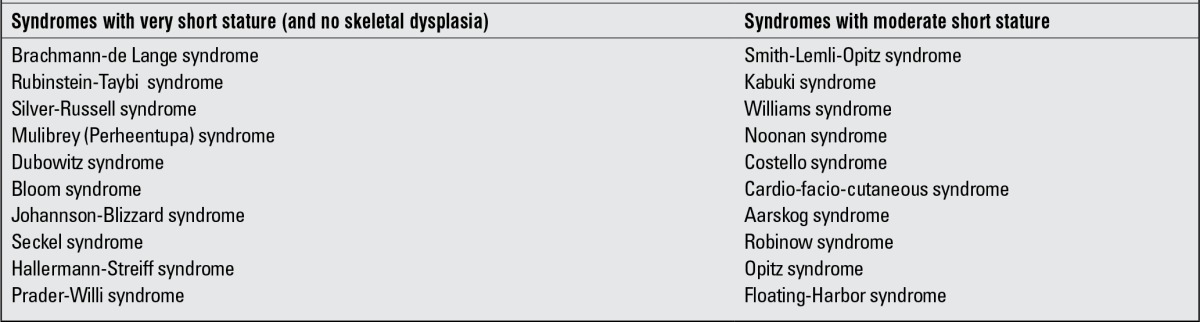
Syndromes associated with short stature

**Table 2:**
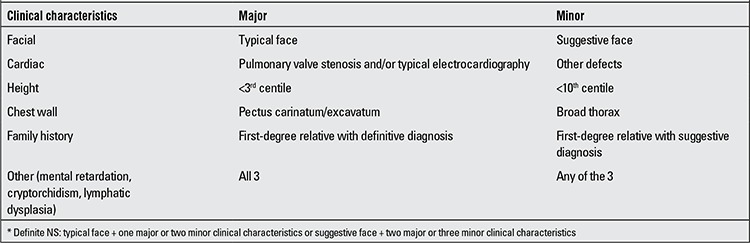
Diagnostic criteria for Noonan syndrome [adapted from Van der Burgt ([ref:8]8[/ref])]

**Table 3:**
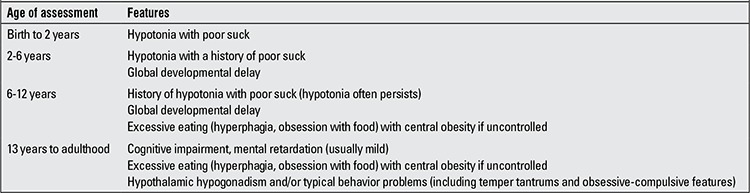
Suggested clinical criteria to prompt DNA testing for Prader-Willi syndrome ([ref:13]13[/ref])

**Table 4:**
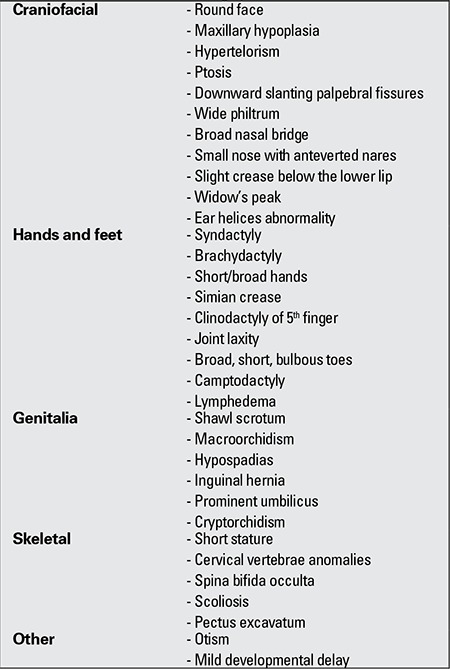
Clinical features in Aarskog syndrome
